# DNase-1 Treatment Exerts Protective Effects in a Rat Model of Intestinal Ischemia-Reperfusion Injury

**DOI:** 10.1038/s41598-018-36198-2

**Published:** 2018-12-12

**Authors:** Shikai Wang, Tian Xie, Shilong Sun, Kai Wang, Baochen Liu, Xingjiang Wu, Weiwei Ding

**Affiliations:** Research Institute of General Surgery, Jinling Hospital, Nanjing University School of Medicine, Nanjing, 210002 Jiangsu Province P.R. China

## Abstract

A growing number of studies have recently revealed a potential role for neutrophil extracellular traps (NETs) in the development of inflammation, coagulation and cell death. Deleterious consequences of NETs have been identified in ischemia-reperfusion (I/R)-induced organ damage, thrombosis and sepsis. And exogenous DNase-I has been suggested as a therapeutic strategy to attenuate ischemia-reperfusion (I/R) injuries in the kidney, brain and myocardium. Herein, we designed a study to investigate whether NETs contribute to the pathogenesis of intestinal I/R injury and evaluated the therapeutic value of DNase-1 in a rat model of intestinal I/R injury. In this rat model of intestinal I/R injury, we found that extracellular DNA was readily detectable in rat serum after 1 h of ischemia and 2 h of reperfusion. Treatment with DNase-1 significantly reduced the inflammatory response, restored intestinal barrier integrity and increased the expression of tight junction proteins. Our results indicate the existence of NETs in I/R-challenged intestinal tissues and firstly provide more evidence that DNase-1 may be an effective treatment for attenuating intestinal I/R injury.

## Introduction

Intestinal ischemia-reperfusion (I/R) injury is a pathophysiologic process during which ischemic intestinal function deteriorates following blood flow restoration^1–3^. The anatomical characteristics of the intestine make it susceptible to ischemia, and reperfusion can enhance such injuries^2,4^. As a driver of systemic inflammatory response syndrome and multiple organ dysfunction syndrome, intestinal I/R can result in increased morbidity and mortality^5,6^. Once an I/R injury has occurred, the integrity of the gastrointestinal barrier is breached, allowing for the translocation of bacteria and endotoxins, and the intestine is thought to be an important target organ during shock, trauma and sepsis^7–9^. Therefore, the prevention and treatment of intestinal I/R and related injuries has become the subject of intense scrutiny.

As the cellular front-line of defense against pathogens, neutrophils play a key role in restricting infections via phagocytosis and degranulation. In 2004, Brinkmann, *et al*.^10^ identified a novel mechanism in which neutrophils create extracellular traps, known as neutrophil extracellular traps (NETs), which comprise chromatin and granule proteins. NETs appear to play a protective role by trapping microorganisms to prevent them from spreading. However, NETs have also been shown to exert harmful effects by triggering coagulation, inflammation and cell death^11,12^. In disease states characterized by the excessive release of NETs, such as sepsis, transfusion-related acute lung injury and rheumatic diseases, these complexes can contribute to disease progression^13–15^. I/R plays a fundamental role in the pathophysiology of several clinical-surgical conditions, and researchers have reported that NETs affect several organs following I/R injury, including the kidneys, brain and myocardial tissues^16–18^. Moreover, DNase-1 treatment can reduce intestinal neutrophil infiltration, indicating that NETs may contribute to the development and progression of intestinal I/R injury. However, a role for NETs in intestinal I/R injury has not been demonstrated.

It has been reported that cell-free DNA (cfDNA) is the principal component of NETs and can be degraded by deoxyribonuclease I (DNase-1)^19^. A retrospective observational study suggested that cfDNA may provide a prognostic utility in patients with severe sepsis and cfDNA has been shown to mediate inflammation and coagulation via multiple mechanisms^20^. Indeed, high levels of cfDNA have been observed in the blood during I/R, inflammation, coagulation and sepsis^13,16,21^, and the administration of DNase-1 is reportedly effective during I/R injury and sepsis^16,18,22^. The successful clearance of NETs in an *in vivo* study showed that DNase-1 can attenuate acute kidney, hepatic and myocardial I/R injury^16,18,23^. Recently, we reported that NETs contribute to intestinal damage in endotoxemic rats and that treatment with DNase-1 may protect the intestine from injury^21^. Although DNase-based therapeutic strategies have been applied in various disease models, the potential therapeutic value of DNase-1 in the physiopathology of intestinal I/R injury remains unknown.

In the present study, we aimed to investigate whether NETs contribute to the pathogenesis of intestinal I/R injury, and found that NETs were highly detected and are concomitant with the intestinal I/R injury. In addition, we firstly evaluated the therapeutic value of DNase-1 in a rat model of intestinal I/R and showed that the degradation of NETs by DNase-1 reduced intestinal I/R-induced epithelial injury and inhibited the loss of natural barrier function.

## Materials and Methods

### Animals

Seventy-five adult male Sprague-Dawley rats, weighing between 180 and 220 g, were purchased from the Experimental Animal Center, Jinling Hospital, Nanjing, China. All experimental procedures were performed in compliance with local and national ethical guidelines and were approved by the Institutional Animal Care and Use Committee at Jinling Hospital, Nanjing University. All the rats were randomized into the following 3 groups: group I (sham group; n = 25), group II (vehicle-control group; n = 25) and group III (DNase-1 treatment group; n = 25).

### Anesthetic and surgical procedures

Prior to surgery, all the rats were housed in a pathogen-free environment with a 12 h light/12 h dark cycle and provided free access to standard rodent food and water. During surgery, the rats were placed on the operation table in a biosafety hood and were anesthetized with chloral hydrate (350 mg/kg b.w.) intraperitoneally. After administration of anesthesia, the subxiphoid area of the rats was shaved and cleaned, the animals underwent a mini-laparotomy, and the intestinal I/R model rats were subjected to warm ischemia for 1 h by clamping the superior mesenteric artery, followed by 2 h of reperfusion under sterile conditions. The DNase-1 treatment group received a single DNase-1 injection (10 mg/kg b.w.;Sigma-Aldrich, St. Louis, MO, USA) intravenously after 1 h of ischemia and 2 h of reperfusion. A bovine-extracted pancreatic DNase I dissolved in saline was used in our research with a solubility of 5.0 mg/ml. The vehicle-control group received a single saline injection (10 mg/kg b.w.) intravenously after the 1 h of ischemia and 2 h of reperfusion. The sham group was subjected to surgery of similar duress and duration without I/R induction. Body temperature was maintained between 36 °C and 37 °C during the surgery. The rats were sacrificed 2 h after intervention. Blood samples and ileum tissues were collected.

### Quantification of serum NET formation

Circulating DNA levels have been reported as a marker of NET formation^19^. We used a capture enzyme-linked immunosorbent assay (ELISA) to quantify the NET levels in the serum as previously described^14^. The commercially available Cell Death Detection ELISA kit (Sigma, 11544675001) and the MPO Mouse ELISA kit (Hycult Biotech, Frontstraat 2a, 5405 PB Uden, The Netherlands) were used to determine the NET concentrations in the serum. The optical density (OD) between 405 and 490 nm was measured to reflect the concentration of cfDNA in the serum. Values for the formation of soluble NETs are presented as the fold induction with the values of the sham group set to 1.

### Identification of NETs *in vivo*

As previously described, CitH3-DNA complexes and MPO can be used as markers to identify NETs and neutrophils in the rat intestine^24^. Thus, we used an immunofluorescence assay to identify NETs and neutrophils *in vivo*. Optimal cutting temperature (OCT) compound-embedded frozen mouse ileum tissues were sectioned (7-μm thickness). Antigen retrieval was performed by boiling the samples for 20 minutes with citrate buffer in a microwave oven. The sections were incubated with an antibody against either Cit-Histone 3 (Proteintech Group, Chicago, USA) or MPO (BOSTER, Wuhan, China),and then incubated with species-specific secondary antibodies coupled with Alexa Fluor 488 Dyes. DNA was stained with 4′,6-diamidino-2-phenyl indole (DAPI). An inverted fluorescence microscope (Olympus Corporation, Tokyo, Japan) was used to image the sections and measure the staining intensity.

### Detection of IL-6, IL-10 and tumor necrosis factor-alpha by ELISA

The IL-6, IL-10 and tumor necrosis factor-alpha (TNF-α) levels in the serum, which reflect the severity of inflammation, were measured using corresponding ELISA kits (KeyGen Biotech, Nanjing, China) according to the manufacturer’s instructions. A standard curve was used to calculate the concentrations of IL-6, IL-10 and TNF-α.

### Morphological analysis of the intestine

Ileal tissues were fixed in 4% buffered paraformaldehyde, embedded in paraffin, and sectioned (4 μm) for histologic analysis. The slides were stained with hematoxylin-eosin and quantitatively assessed according to the Chiu score system by three pathologists who were blinded to the treatment conditions.

### Western blotting analysis

Tight junction proteins (TJPs), including claudin-1, occludin and ZO-1, play a crucial role in maintaining the functional integrity of the intestinal mucosa barrier. Thus, Western blot analysis was performed to determine the expression levels of the TJPs.

Proteins extracts from the ileums were resolved by SDS-PAGE and transferred to PVDF membranes(KeyGen Biotech, Nanjing, China). The membrane wasblocked in blocking buffer (KeyGen Biotech, Nanjing, China), followed by incubation with corresponding primary antibodies(KeyGen Biotech, Nanjing, China). After the membranes were washed three times, they were incubated with secondary antibodies (KeyGen Biotech, Nanjing, China) for 1 h at room temperature. Protein bands were visualized using an enhanced chemiluminescence (ECL) Western blotting detection reagent and imaged using a G:BOX ChemiXR5 imaging system. The band intensities were analysed using Gel-Pro32 software.

### Immunofluorescence

Immunostaining was performed as previously described^25^. The ileum tissues were rinsed in PBS and fixed with 2.5% paraformaldehyde. Then, the collected tissues were embedded in OCTcompound and frozen prior to slicing 5-μm-thick cryosections. The slides were blocked with normal goat serum in PBS for 20 minutes before they were incubated with monoclonal antibodies targeting claudin-1 (KeyGen Biotech, Nanjing, China), occludin (Proteintech Group, Chicago, USA) and ZO-1 (Proteintech Group, Chicago, USA). After the sections were washed three times with PBS, they were incubated with secondary antibodies (Jackson Immuno Research Laboratories, Inc, West Grove, PA, USA) for 1 h. The sections were subsequently counterstained with DAPI. Images were captured using a microscope (Olympus Corporation, Tokyo, Japan).

### Statistical analysis

All data are expressed as the means ± SEM. Differences between groups were determined using one-way ANOVA followed by the Student–Newman–Keuls test. P values < 0.05 were considered significant. All statistical analyses were performed using the GraphPad Prism (version 5.01; GraphPad Software, Inc, La Jolla, CA).

## Results

### Detection of NET formation after intestinal I/R injury

**Figure 1:**
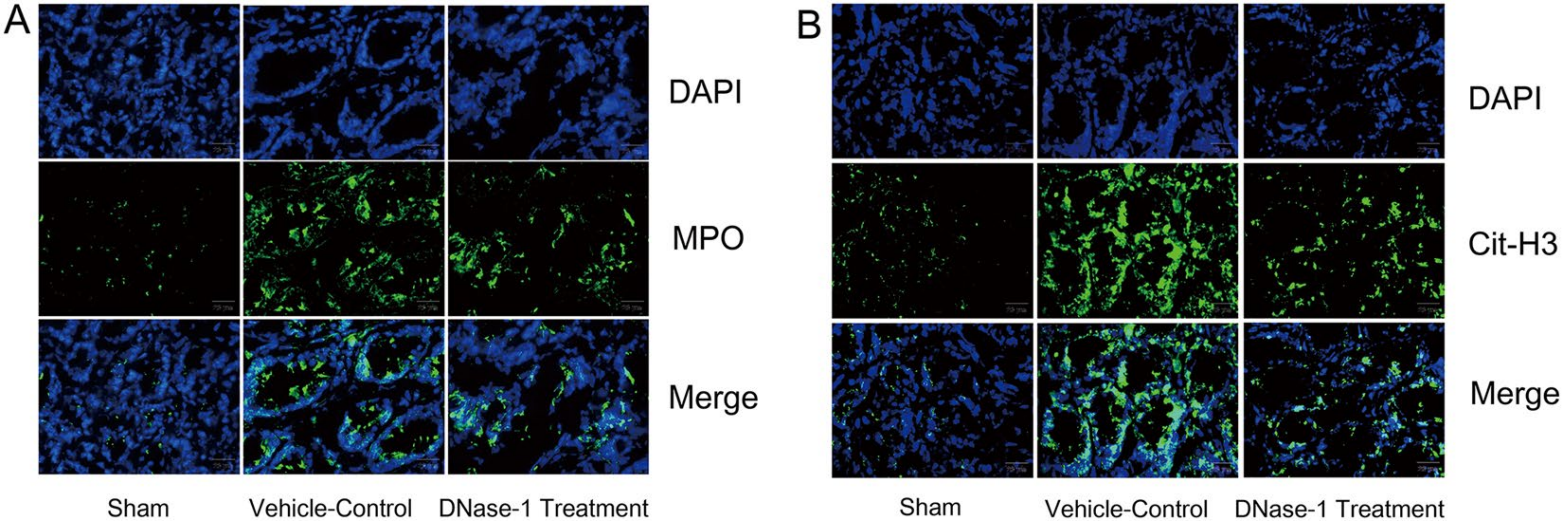
Identification of NETs *in vivo* by immunofluorescence staining. (**A**) Rats were subjected to intestinal I/R injury to induce neutrophil infiltration (MPO positive, green, n = 4 per group). Scale bar: 20 μm. (**B**) NETs were detected after 1 h ischemia and 2 h reperfusion (Cit-H3-positive, green, n = 4 per group). Scale bar: 20 μm.

**Figure 2:**
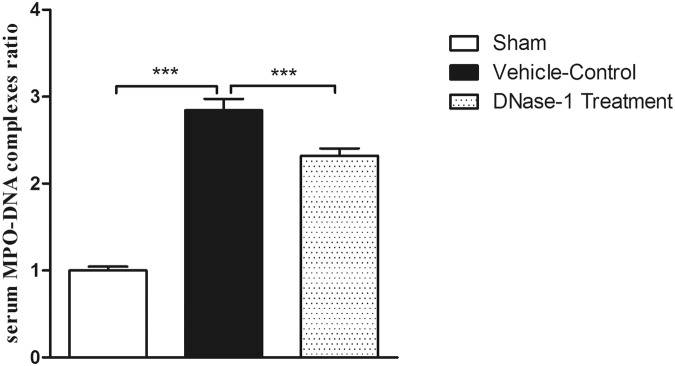
Quantitation of serum cell-free DNA in between groups using capture enzyme-linked immunosorbent assay (ELISA). Increased NET formation was detected in the vehicle-control groups compared with the sham group (***P < 0.001). DNase-1 intervention significantly decreased NET formation (**P < 0.01 versus the vehicle-control group). Data are expressed as mean ± SEM (n = 15–20 per group).

To investigate NET formation in the intestine during I/R injury, immunofluorescence staining was performed. As shown in Fig. 1, we observed a significant neutrophil infiltration (MPO positive) after intestinal I/R injury. NETs (Cit-H3 positive) were also observed in the ileum tissue during intestinal I/R injury, whereas fewer NETs were detected in the sham group. Notably, we detected lower NET levels following DNase-1 intervention. cfDNA has been used as a marker for NETs in the serum. Therefore, we used ELISA to quantify NET levels in our study. As shown in Fig. 2, cfDNA levels in the I/R control group were elevated, whereas cfDNA levels were significantly reduced following intravenous injection of DNase-1(P < 0.01). These results indicate that NETs are present in the intestine and that cfDNA is released into the blood during intestinal I/R injury. Furthermore, these results suggest that treatment with DNase-1 significantly disrupts existing NETs and decreases the formation of NETs in the intestine and serum during intestinal I/R injury.

### DNase-1 treatment reduces the early proinflammatory response after intestinal I/R injury

**Figure 3:**
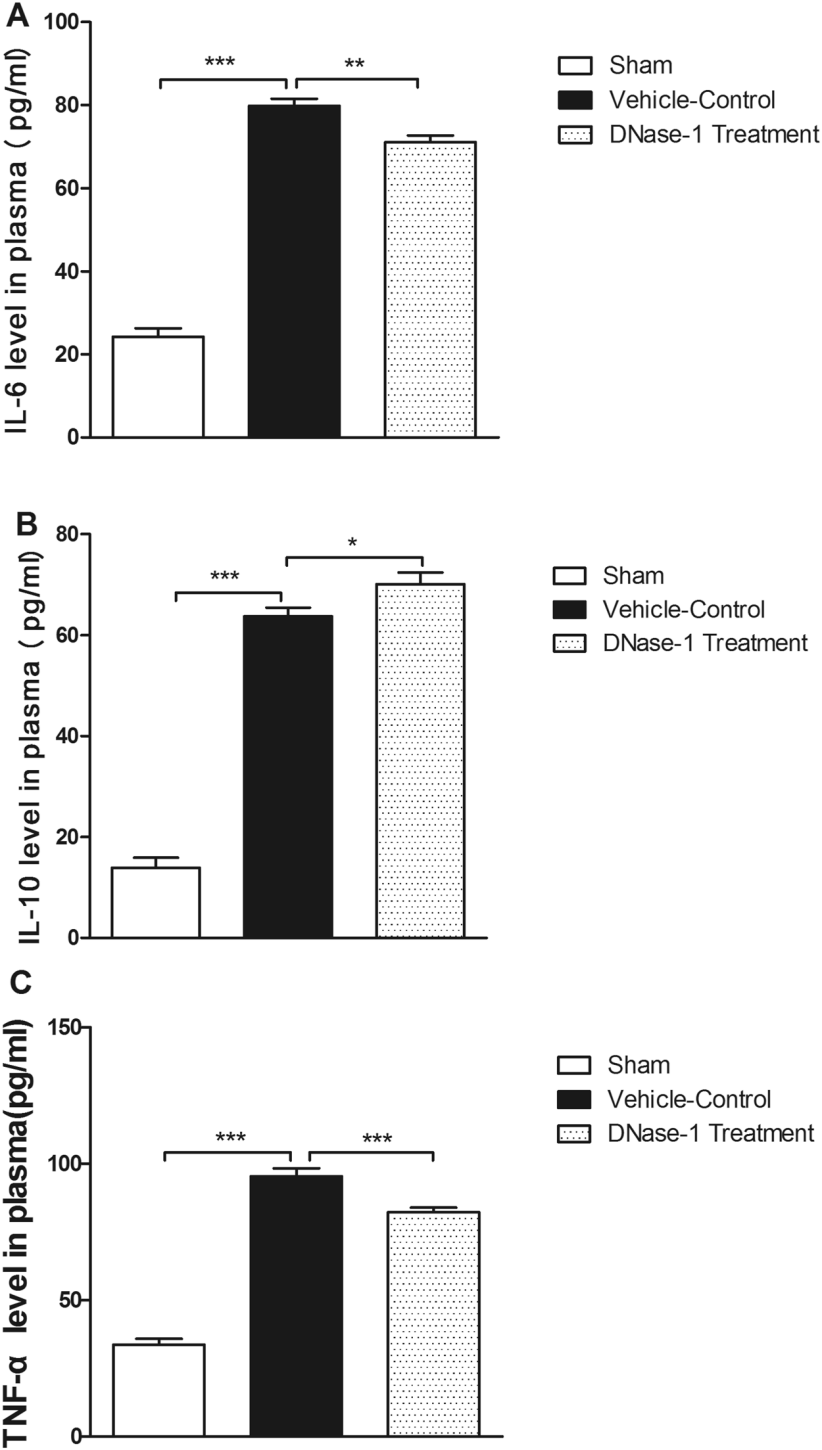
A comparison of serum inflammatory cytokine expression between groups as assessed by ELISA. Levels of inflammatory cytokines were significantly increased after intestinal I/R injury. DNase-1 intervention decreased the levels of proinflammatory cytokine IL-6 and TNF-α, and increased the levels of anti-inflammatory cytokine IL-10. (**A**) IL-6, (**B**) IL-10 and (**C**) TNF-α. The values are presented as the means ± SEM (n = 15–20 per group, *P < 0.05, **P < 0.01 and ***P < 0.001).

Based on our observation that intestinal I/R injury results in the excessive release of NETs, we decided to investigate the relationship between NET degradation and the early inflammatory response. Therefore, we measured levels of the proinflammatory cytokines IL-6, TNF-α and the anti-inflammatory cytokine IL-10 using ELISA. As shown in Fig. 3, compared with the control group, the IL-6 and TNF-α levels were significantly decreased in the DNase-1 treatment group (P < 0.01 and P < 0.001, respectively). Interestingly, we also found that the anti-inflammatory cytokine IL-10 was increased in the treatment group (P < 0.05) compared with the controls. Taken together, these results suggest that NETs contribute to the early inflammatory response after intestinal I/R injury and that DNase-1 may attenuate the proinflammatory response associated with NETs.

### DNase-1 treatment attenuates histopathological changes after intestinal I/R injury

**Figure 4:**
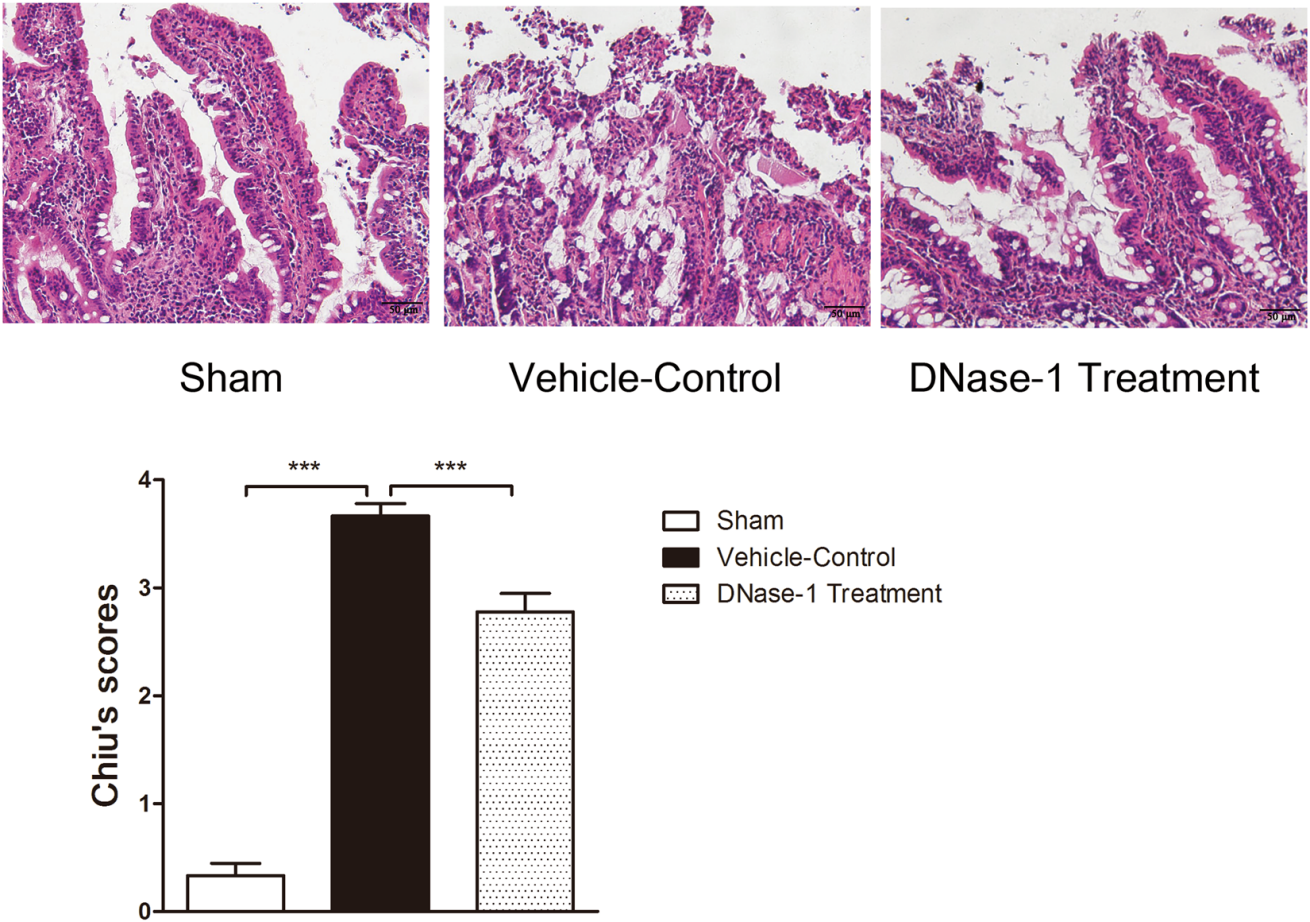
Comparisons of the histopathological changes among groups using hematoxylin-eosin staining and the Chiu scoring system. The DNase-1 treatment group showed a higher score than the vehicle-control group (n = 6 per group, mean injury score: 2.778 vs. 3667, ***P < 0.001).

Hematoxylin-eosin staining was performed to identify intestinal histopathological changes after I/R injury. As shown in Fig. 4, the villi of the intestinal mucosa exhibited normal morphology in the sham group, whereas the vehicle-control group showed extensive damage to the intestinal epithelium, including villous desquamation and inflammatory cell infiltration. However, rats treated with DNase-1 showed relatively minor injuries compared with the vehicle-control group. The severity of the intestinal mucosa injuries was also graded according to the Chiu scoring system. The DNase-1 treatment group had a lower score than the vehicle-control group (mean injury score: 2.778 vs. 3.667, P < 0.001). Taken together, these results suggest that the DNase-1 intervention can inhibit histopathological changes after intestinal I/R injury.

### DNase-1 treatment ameliorates the disruption of tight junction proteins

**Figure 5:**
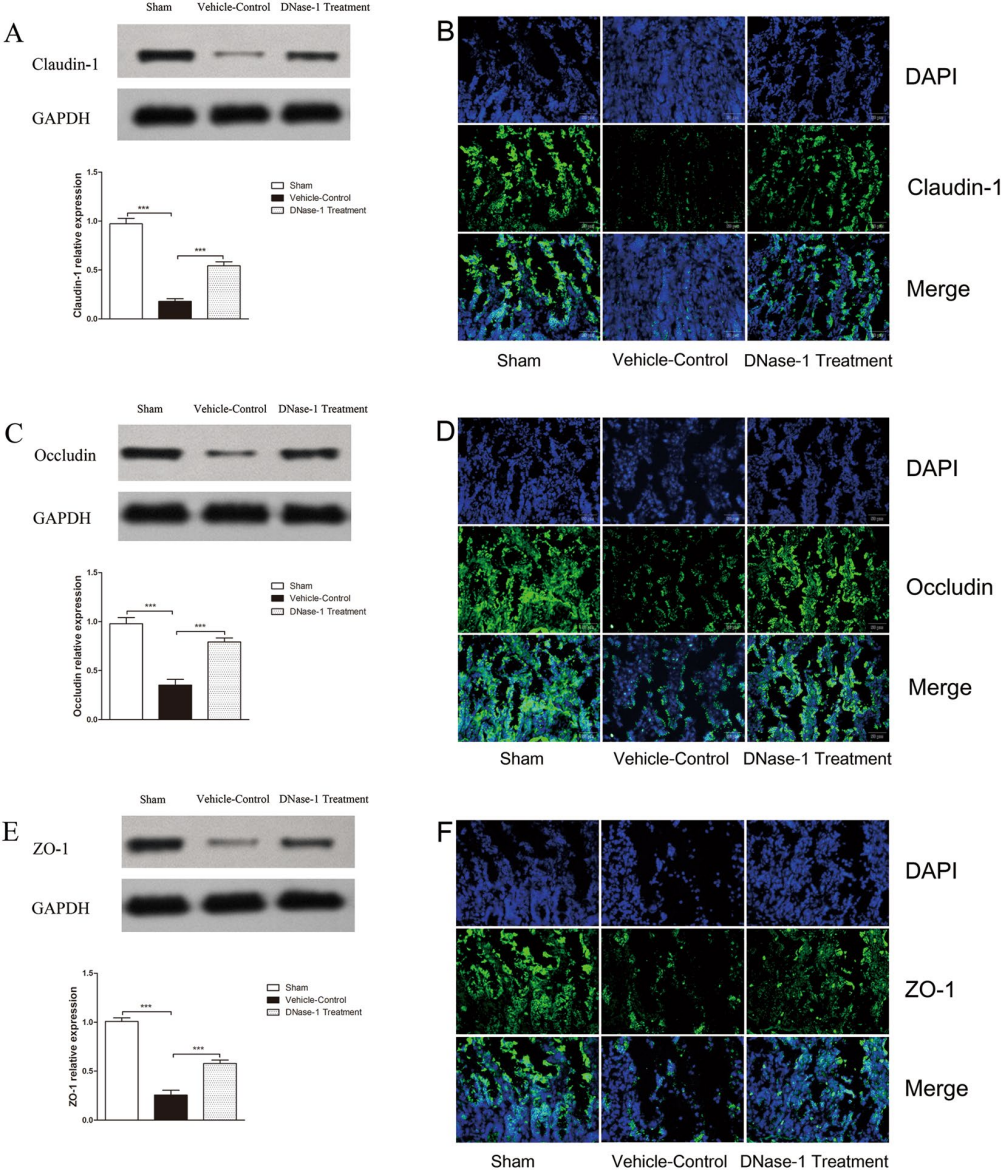
A comparison of tight junction protein expression between groups. (**A**) Western blotting analysis claudin-1 protein expression (Full data about WB were available in the Supplementary information). The vehicle-control group had reduced claudin-1 concentrations compared with the sham group (***P < 0.001). The DNase-1 treatment group had increased claudin-1 levels compared with the vehicle-control group (***P < 0.001). (**B**) Immunofluorescence analysis of claudin-1 (green) and DAPI (blue). (**C**) Western blotting analysis of occludin. Compared with the vehicle-control group, DNase-1 intervention effectively increased occludin expression (*** P < 0.001). (**D**) Immunofluorescence analysis of occludin (green) and DAPI (blue). (**E**) Western blotting analysis ZO-1 protein expression. DNase-1 intervention significantly increased the expression of ZO-1 (***P < 0.001) compared with the vehicle-control group. (**F**) Immunofluorescence analysis of ZO-1 (green) and DAPI (blue). All data are presented as mean ± SEM (n > 6 per group). Scale bar: 50 μm.

As a major component of the epithelial barrier, it is generally accepted that TJPs seal the paracellular space between adjacent epithelial cells to exclude the outside environment. Therefore, we visualized the expression of several TJs, including claudin-1, occludin and ZO-1 and used Western blot analysis and immunofluorescence to assess the integrity of the epithelial barrier. As shown in Fig. 5, we observed obvious improvements in TJP levels in the DNase-1 treatment group compared with the vehicle-control group. The relative expression of the TJPs was also measured and the results showed a significant difference between the vehicle-control group and the DNase-1 treatment group. The results indicate that the DNase-1 intervention can significantly restore intestinal barrier structures and the expression of TJPs.

## Discussion

Epithelial injury and loss of natural barrier function due to intestinal I/R^5^, which can lead to neutrophil infiltration, are hallmarks of “NETosis”^26^. Here, we report for the first time the existence of NETs in the rat intestine during I/R injury, and show that DNase-based interventions could reduce NET density, downregulate the proinflammatory response, attenuate intestinal histological changes and maintain the functional integrity of tight junctions and the cytoskeleton. Therefore, our results provide experimental evidence for the therapeutic potential of NET-targeted interventions in intestinal I/R injury. Based on our observations and other recent publications, we here propose plausible mechanisms and therapeutic options for NET-mediated intestinal I/R injury treatments.

### Cell-free DNA levels increase after intestinal I/R injury

A large number of studies on NETs have been published over the past decade, and elevated levels of cfDNA, which is present in various pathologic states, have been consistent markers for immune responses to inflammatory and coagulation conditions^20^. Once intestinal I/R injury occurs, large numbers of neutrophils infiltrate the intestinal tissues, followed by NETosis – a newly described form of active cell death that results in the copious release of cfDNA into the bloodstream. In our study, we found that extracellular DNA was readily detectable in rat serum and ileum tissue after 1 h of ischemia and 2 h of reperfusion. Several previous studies about I/R injuries corroborate our results, however none included the intestine. Simon *et al*. reported that hypoxia elevated circulating levels of nucleosomes and stroke increases extracellular chromatin markers of cerebral I/R injury^17^.

In this study, we observed that intestinal I/R injury leads to the copious release of cfDNA into the bloodstream. Concurrently, we detected neutrophil infiltration and NET formation in the intestinal tissue. These observations therefore provide substantially greater evidence that NETs are associated with intestinal I/R injury. As previously reported, NETs are mainly derived from neutrophils^10^; however, as I/R is occurring, intestinal epithelial cells are also undergoing apoptosis. Therefore, identifying the origin of circulating DNA released during intestinal I/R will likely be complex. Interestingly, our research group previously reported that NETs could induce intestinal cell apoptosis in a CLP rat model^21^. Therefore, although the origin of this DNA may remain obscure, cfDNA may be a marker for intestinal I/R injury.

Endogenous cfDNA levels may contribute to intestinal I/R injury. Elevated cfDNA levels are associated with the severity of injury and the clearance of cfDNA can reduce intestinal tissue damage. Gould TJ and colleagues demonstrated that extracellular DNA could drive disease progression via multiple mechanisms involving coagulation, inflammation, and cell death^12^, Similar with the previous studies, we provided solid evidences that circulating cfDNA functions not only as a marker of intestinal I/R injury but also as a major causative agent that can aggravate disease progression.

### A DNase-based intervention is protective after intestinal I/R injury

Previous studies have shown that DNase-1 can degrade cfDNA, a major structural constituent of NETs, thereby limiting the extent of injury caused by NETs during sepsis, thrombosis and I/R^27^. And Boettcher et. elucidated the therapeutic efficacy of DNase-1 on ameliorating tissue injury in the intestine^28^. However, we firstly evaluated the therapeutic effect of DNase-1 on preserving the integrity of the intestinal mucosal barrier during I/R. We hypothesized that DNase-1 may have beneficial impacts on intestinal I/R injury and verified whether NET degradation resulted in improved intestinal microcirculation under our experimental conditions. Based on a previous reports^16^, we chose a dose of 0.1 mg/kg b.w. in this study and a pilot study^29^, was used as a basis for the DNase-1 administration schedule (i.e., 2 h after surgery). Encouragingly, all the assessed parameters of systemic inflammation (serum IL-6, IL-10 and TNF-α), intestinal epithelium injury and histopathological changes were improved following intervention compared with the vehicle-control groups, indicating that NETs may accelerate intestinal I/R injury and that DNase-1 can attenuate these effects. Meanwhile, we observed a significant decrease in cfDNA serum levels following treatment with DNase-1. Furthermore, significant correlation between cfDNA levels and intestinal epithelium injury was found, consistent with previous findings^20^. Therefore, our results provide further evidence for a detrimental role for NETs in intestinal I/R injury.

NETs comprise neutrophil DNA, granules and antimicrobial proteins, including histones, neutrophil elastase, MPO, pentraxin (PTX), lactoferrin, cathepsin G and bactericidal permeability-increasing protein^27^. The increasing roles of NETs in various disease conditions have heightened the importance of NET-targeted treatments. We found that NETs are involved in the development and progression of intestinal I/R injury. Thus, a plausible treatment strategy is to degrade this complex, and encouragingly, our results demonstrate that such treatments can be effective. However, whether other therapeutic options targeting histones, neutrophil elastase or other NET components be effective requires further investigation. Additionally, whether the different dose of DNase-1 may exert a different outcome on intestinal I/R remain poorly understood. Thus, the dose and timing of treatment initiation warrants further analysis. It is our hope that more evidence-based therapeutic options will become available in the near future. Our research focus on exploring the effect of short time administration of DNAse-1 on rats after 1 h of ischemia and 2 h of reperfusion. However, the long-term outcome of DNase-1 treatment remains obscure. Moreover, the influence of DNAse-1 on intestine subjected to different reperfusion times after injury by 1 h ischemia is unknown. It would be very interesting to understand these issues in future work.

### NETs exacerbate the intestinal inflammation after I/R

As a driver of systemic inflammatory response syndrome and multiple organ dysfunction syndrome, the intestine is critical in immune system function. Notably, an absolute intestinal mucosal barrier provides an essential defense against external pathogens; however, there is no universally accepted theory to define the mechanisms responsible for the initiation and development of intestinal I/R injury to date^30^. Another related issue is the fact that the clinical management of intestinal I/R injury is challenging because of a limited knowledge of the underlying pathophysiology.

Our understanding of NETs has substantially progressed since Brinkmann *et al*. first proposed the specific role of neutrophils. However, previous publications on intestinal I/R injury have not explored this unique mechanism. Likewise, the role by which NETs exert their effects during intestinal I/R injury is complex and unclear. Therefore, we designed this study to identify the role of NETs in the injury response and explore whether they could provide a new mechanistic insight into this common disease. Encouragingly, our study found that NETs were involved in intestinal I/R injury; thus, we postulated the potential role of NETs in intestinal I/R injury.

An increasing number of reports on the association of NETs with inflammation, have recently been published^11,13,31^. In our study, we found increased NET levels following injury. NETs could damage epithelial and endothelial cells^14^, which exacerbated I/R-induced intestine injury. NETs may lead to the inflammatory response by impairing the intestinal epithelial cells, and our study demonstrated that the functional integrity of tight junctions and the cytoskeleton structure was destroyed after intestinal I/R injury. Once the bacteria and endotoxins were released into systemic circulation, a systemic proinflammatory response may ensue, as observed in our study. Therefore, NETs contribute to the intestinal inflammation after I/R injury.

A better understanding of the contribution of NETs to injury will assist in guiding treatment decision-making. Recent reports have suggested that NETs may contribute to the coagulation and cell death^12,21^. Further studies are needed to elucidate the therapeutic efficacy of combinations of antimicrobial agents, anticoagulant therapy and NET-targeted interventions.

## Electronic supplementary material

Supplementary information
