# Supplementary material for: DNase-1 Treatment Exerts Protective Effects in a Rat Model of Intestinal Ischemia-Reperfusion Injury
Source: Sci Rep. 2018 Dec 12;8:17788. doi: 10.1038/s41598-018-36198-2 (PMC6290768; doi:10.1038/s41598-018-36198-2)
Supplement: Supplementary file 1 — Supplementary information [file 41598_2018_36198_MOESM1_ESM.pdf]

DNase-1 Treatment Exerts Protective Effects in a Rat Model of Intestinal Ischemia-Reperfusion Injury

Shikai Wang[1,*], Tian Xie [1,*], Shilong Sun[1], Kai Wang[1], Baochen Liu[1], Xingjiang Wu[1], Weiwei Ding[1]

[1]Research Institute of General Surgery, Jinling Hospital, Nanjing University School of Medicine, Nanjing 210002, Jiangsu Province, P.R. China.

*These authors contributed equally to this work.

Correspondence and requests for materials should be addressed to W.D. (e-mail: dingwei_nju@hotmail.com)

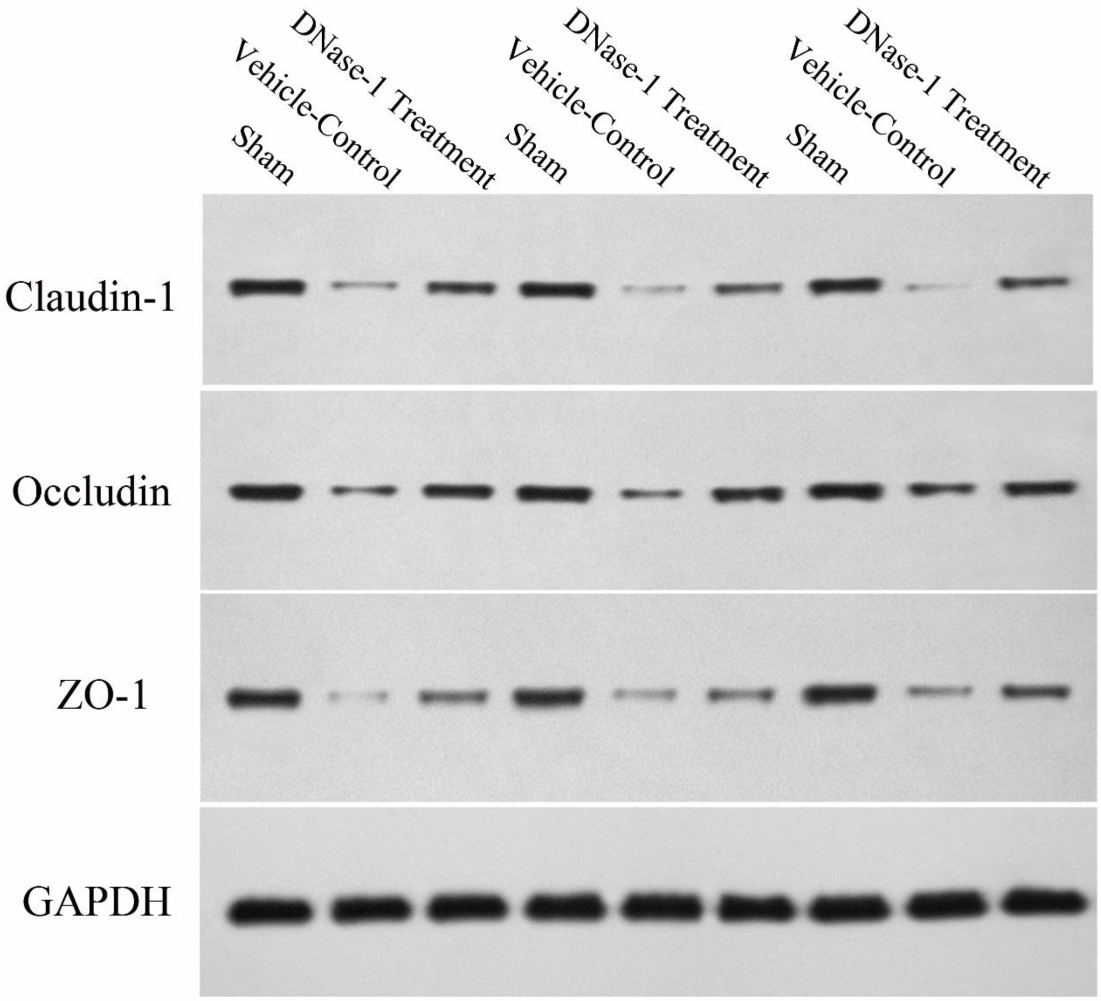

Full-length blots of claudin-1, occluding and zo-1 between groups.
